# Circular RNA hsa_circ_0069117 suppresses proliferation and migration of osteosarcoma cells lines via miR-875-3p/PF4V1 axis

**DOI:** 10.1186/s13018-022-02923-x

**Published:** 2022-01-21

**Authors:** Ziyan Zhang, Lin Zhou, Shicheng Zhou, Xin Li

**Affiliations:** Orthopedic Medical Center, the Second Hospital of Jilin University, Ziqiang Street 218, Changchun, 130041 Jilin China

**Keywords:** Osteosarcoma, hsa_circ_0069117, miR-875-3p, PF4V1, Cell proliferation and migration

## Abstract

**Background:**

Osteosarcoma (OS) is one of the most common malignant bone tumors in children and adolescents. Circular RNAs (circRNAs) are critical regulators involved in multiple physiological and pathological processes. However, the underlying regulatory mechanisms of circRNA in OS are still not fully understood.

**Methods:**

The circRNA expression profiles were downloaded from the Gene Expression Omnibus (GEO) database and analyzed by GEO2R. Bioinformatics analysis was performed to predict the potential target miRNAs of hsa_circ_0069117 and its downstream mRNAs. The co-expression of hsa_circ_0069117/miR-875-3p/PF4V1 axis was further validated in OS tissue samples via quantitative real-time PCR (qRT-PCR). Luciferase reporter gene plasmids containing the sequence of PF4V1 and hsa_circ_0069117 were constructed to verify the putative sites of miR-875-3p. Gain/loss-of-function assays were performed to verify the effect of hsa_circ_0069117 on miR-875-3p/PF4V1 expression and related pathways via qRT-PCR and Western blot. Cell counting kit-8 (CCK-8) and wound-healing assays were performed to evaluate the effect of hsa_circ_0069117 on cell proliferation and migration of MG63 and U2OS, respectively.

**Results:**

We identified hsa_circ_0069117 as the most markedly dysregulated circRNA in OS cell lines. Bioinformatics analysis indicated that hsa_circ_0069117 might inhibit the expression of miR-875-3p, thereby promoting the expression of platelet factor 4 variant 1 (PF4V1). The expression of miR-875-3p was negatively correlated to hsa_circ_0069117 and PF4V1 in clinical samples. Luciferase reporter gene assays confirmed the binding sites of miR-875-3p on hsa_circ_0069117 and PF4V1. Gain/loss-of-function and rescue assays further indicated that hsa_circ_0069117 could significantly promote the expression of PF4V1 by sponging miR-875-3p, thereby inhibiting the proliferation and migration of OS cells by suppressing ERK1 and AKT.

**Conclusion:**

Our study revealed that hsa_circ_0069117 is an anti-OS molecule that could substantially attenuate cell proliferation and migration of OS, which may provide a novel and reliable molecular target for the treatment of OS patients.

## Introduction

Osteosarcoma (OS) is the most common primary malignant bone tumor accounting for 10% of solid tumors in children and adolescents [1, 2]. It has been characterized as a highly fatal disease with strong invasiveness and a high metastasis rate [2]. Aggressive chemotherapy plus surgery is the primary therapeutic method for OS with an overall 5-year survival rate of 70–80% [3–5]. However, patients with lung metastases still have a low 5-year survival rate of 5–10% and a high recurrent rate of 20–30% [3]. Although recent decades have witnessed significant progress in molecular targeting therapy of OS [6, 7], few advances have been made in promoting survival rate during the last 25 years, especially for metastatic disease [3]. Thus, further insight into the regulation mechanism and new therapeutic approaches of OS are urgently needed to address this problem.

Circular RNAs (circRNAs) were discovered decades ago [8, 9]. It is a type of ncRNA (non-coding RNA) that has been suggested to act as an essential biomolecule in regulating multiple progression of diseases [10, 11]. Circular RNAs have a unique feature to resist most RNases, enabling them to be ideal biomarker for detecting human diseases [9, 12]. It has been proven that circRNAs can bind miRNAs and proteins thereby regulating multiple physiological and pathological processes [13]. Preliminary studies have revealed that some circRNAs are dysregulated in OS, resulting in changes in proliferation, invasion, migration, metastasis, apoptosis, adhesion and multi-drug resistance of OS cells [14, 15].

In the present study, we analyzed the GEO dataset GSE96964 and found that circRNA hsa_circ_0069117, a circular transcription of TBC1 domain family member 14 (TBC1D14), was most markedly dysregulated in OS cells [16]. However, the regulatory effect of hsa_circ_0069117 remains unclear. We validated its expression in OS cell lines and tissues, and further demonstrated that hsa_circ_0069117 could significantly promote the expression of PF4V1 by sponging miR-875-3p, thereby promoting the expression of cell proliferation and migration of OS by upregulating extracellular signal-regulated kinase 1 (ERK1) and protein kinase B (AKT).

## Materials and methods

### Differentially expression analysis of circRNA and miRNA in OS

The circRNA and miRNA expression profiles of OS were downloaded from the Gene Expression Omnibus (GEO) database of the National Center of Biotechnology Information (NCBI, http://www.ncbi.nlm.nih.gov/geo/). The raw data of circRNA and miRNA expression profiles were then analyzed via GEO2R (https://www.ncbi.nlm.nih.gov/geo/geo2r/), an interactive web tool that allows users to identify genes or ncRNAs expressed differentially across experimental conditions [16, 17]. The differentially expressed miRNAs were screened based on criteria of |fold change (FC)|≥ 2 and adjusted* p* < 0.05.

### Target prediction

The target miRNAs of hsa_circ_0069117 were predicted using circBank (http://www.circbank.cn/searchCirc.html). The Functional Enrichment analysis tool (FUNRICH) was used to screen out the potential target mRNAs of miR-875-3p in miRDB, TargetScan, miRWalk, TargetMiner, and microT-CDS databases [18].

### Cell culture

Human osteosarcoma cell lines (OSCL) U2OS, 143B, MG63, HOS, and human mesenchymal stem cell line hMSCs were obtained from the American Type Culture Collection (ATCC). These cell lines were cultured in Dulbecco’s minimum essential medium (DMEM, Gibco, Shanghai) mixed with 10% fetal bovine serum (FBS, Gibco, Shanghai) and 1% antibiotics (streptomycin and penicillin, Gibco, Shanghai). The medium was replaced every 2–3 days.

### Clinical tissues

A total of 6 patients who were diagnosed with conventional osteosarcoma and underwent surgical resection at the Second Hospital of Jilin University were involved in our study. The diagnosis of OS was confirmed by pathological analysis. None of the patients received radiotherapy or chemotherapy before surgery. Osteosarcoma tissues (OST) and paired adjacent normal tissues were obtained after surgery and stored in liquid nitrogen. All the patients involved in this study provided written informed consents. The ethics committee of the Second Hospital of Jilin University approved this study (NO. 2016.169).

### RNA preparation and quantitative real-time PCR analysis

Total RNA was extracted using TRIzol (Invitrogen) following the manufacturer’s instructions. The cDNAs were synthesized using First-Strand Synthesis Kit (TAKARA, Tokyo, Japan). The primers of circRNA, miRNA and mRNA were synthesized by GenePharma (Suzhou, China). Quantitative real-time PCR (qRT-PCR) was performed using the TB Green™ Kit (TAKARA, Tokyo, Japan). The expression of circRNA and mRNA were normalized relative to GAPDH, and miRNAs was normalized relative to U6, which was calculated using 2^−△△Ct^ method. The primers used in this study are listed in Table 1.

**Table 1:** Primers used in this study.

Gene name	Forward (5′–3′)	Reverse (5′–3′)
hsa_circ_0069117	TGCGCATTTCAAGAAGAACA	TCCACTTCAGAGCCTCCTGT
miR-875-3p	CGCGCGCCTGGAAACACTGAG	ATCCAGTGCAGGGTCCGAGG
U6	CGCTTCGGCAGCACATATAC	TTCACGAATTTGCGTGTCATC
PF4V1	GCCAGGAGATGCTGTTCTTG	GGGAGGTGGTCTTCACACAC
AKT	AGCGACGTGGCTATTGTGAAG	GCCATCATTCTTGAGGAGGAAGT
ERK1	TACACCAACCTCTCGTACATCG	CATGTCTGAAGCGCAGTAAGATT
GAPDH	GCACCGTCAAGGCTGAGAAC	TGGTGAAGACGCCAGTGGA

### Luciferase reporter gene assay

The 3′-UTR of PF4V1 and hsa_circ_0069117 containing the putative binding site (wide and mutated type) of miR-875-3p were inserted into pGL6-miR vectors which were then validated by sequencing (Genepharma, Suzhou, China). The 293 T cells were co-transfected by reporter vectors and miR-875-3p mimics (GenePharma, Suzhou, China). Firefly luciferase activity was measured at 48 h after transfection.

### Cell transfection

The hsa_circ_0069117 overexpression vector (ov-hsa_circ_0069117) and siRNA (si-hsa_circ_0069117) were designed and purchased from Hanbio (Shanghai, China). The miR-875-3p inhibitor and mimics were purchased from GenePharma Gene (Suzhou, China). Lipofectamine™ 3000 Transfection Reagent (Invitrogen, Thermo Fisher Scientific Inc., Shanghai, China) was used as the transfection vehicle. The expression of hsa_circ_0069117, miR-875-3p and mRNAs was detected at 48 h post-transfection using quantitative real-time PCR.

### Western blot

Proteins were separated by gel electrophoresis and transferred to a PVDF membrane. The PVDF membrane was incubated with primary antibodies against PF4V1, ERK1, p-ERK1, AKT, p-AKT, and GAPDH (Abcam, Shanghai, China) overnight at 4 °C. Secondary antibodies (Abcam, Shanghai, China) were used to incubate the blots. The ECL luminescence reagent (Invitrogen, Thermo Fisher Scientific Inc., Shanghai, China) was then used to visualize the bands. The quantification analysis of protein was performed for PF4V1, ERK1, p-ERK1, AKT by normalizing to GAPDH.

### Cell proliferation

The effect of hsa_circ_0069117 on MG63 and U2OS proliferation was evaluated using the cell counting kit-8 (CCK-8) assay. OS cells were seeded in 96-well plates and cultured to 80–90% density. CCK-8 solution was added to the cell culture plates at 24 h and 48 h (for 2 h) after hsa_circ_0069117 vector transfection. The optical density was measured at a wavelength of 450 nm to reflect the effect of hsa_circ_0069117 on MG63 and U2OS proliferation.

### Cell migration

The effect of hsa_circ_0069117 on MG63 and U2OS cell migration was evaluated using a wound-healing assay. OS cells were seeded in 96-well plates and cultured to 80–90% density. A straight wound was created manually with a clean 100 μL plastic pipette tip post hsa_circ_0069117 vector transfection. The cell migration areas were recorded by microscopy at 24 h and 48 h post hsa_circ_0069117 vector transfection.

### Data analysis

The expression data were using the t-test to compare differences between two groups. One-way ANOVA was used to compare the differences among multiple groups and *p* < 0.05 indicated that the difference was statistically significant. |FC|≥ 2 and adjusted *p* < 0.05 were set as the threshold for detecting dysregulated circRNAs and miRNAs. Bar graphs were constructed using GraphPad Prism 7.0. The ceRNA network was generated using Cytoscape software.

## Results

### CircRNA hsa_circ_0069117 was most markedly dysregulated in OS cells and predicted to competitively binding to miR-875-3p with PF4V1

**Fig. 1:**
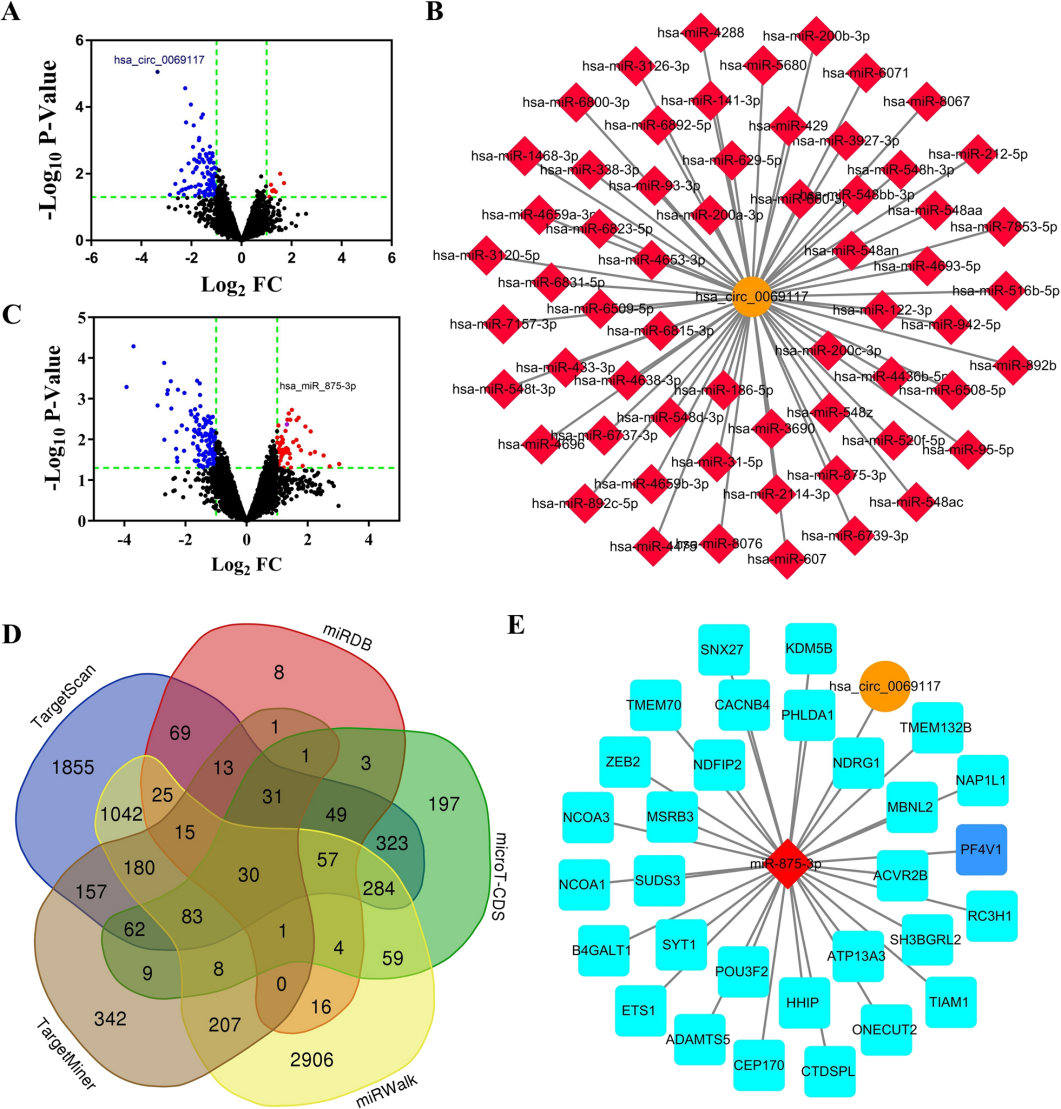
**A** The volcano plots of circRNA expression profile. **B** The regulatory network of hsa_circ_0069117 and its potential target miRNAs. **C** The volcano plots of miRNA expression profile. **D** Venn diagram showed there were 30 potential target mRNAs of miR-875-3p. **E** The ceRNA networks showed the hsa_circ_0069117/miR-875-3p/mRNAs regulatory networks

The circRNA expression profile (GSE96964) and miRNA expression profile (GSE70367) were analyzed using GEO2R to detect the dysregulated circRNAs and miRNAs in OS. According to the criteria, eight upregulated and 102 downregulated circRNAs were screened out (Fig. 1A). A total of 58 upregulated and 126 downregulated miRNAs were detected in GSE70367 (Fig. 1B). Among these differentially expressed circRNAs, hsa_circ_0069117 was the most markedly dysregulated in OS cells lines (fold change: − 10.24, *p* value: 0.00000881). Next, we predicted 60 potential target miRNAs of hsa_circ_0069117 (Fig. 1C). However, among these targeted miRNAs, only miR-875-3p was dysregulated in OS with a fold change of 2.50 and a *p* value of 0.0042548. Venn analysis indicated that 30 mRNAs were predicted to be miR-875-3p targets (Fig. 1D). The ceRNA network was constructed to show the potential relationship between hsa_circ_0069117, miR-875-3p, and target mRNAs (Fig. 1E). Then, we performed a literature review and found that PF4V1 has already been validated as miR-875-3p target, which was also dysregulated in several tumor diseases [19].

### The expression of hsa_circ_0069117, miR-875-3p, and PF4V1 was correlated in OS tissues

**Fig. 2:**
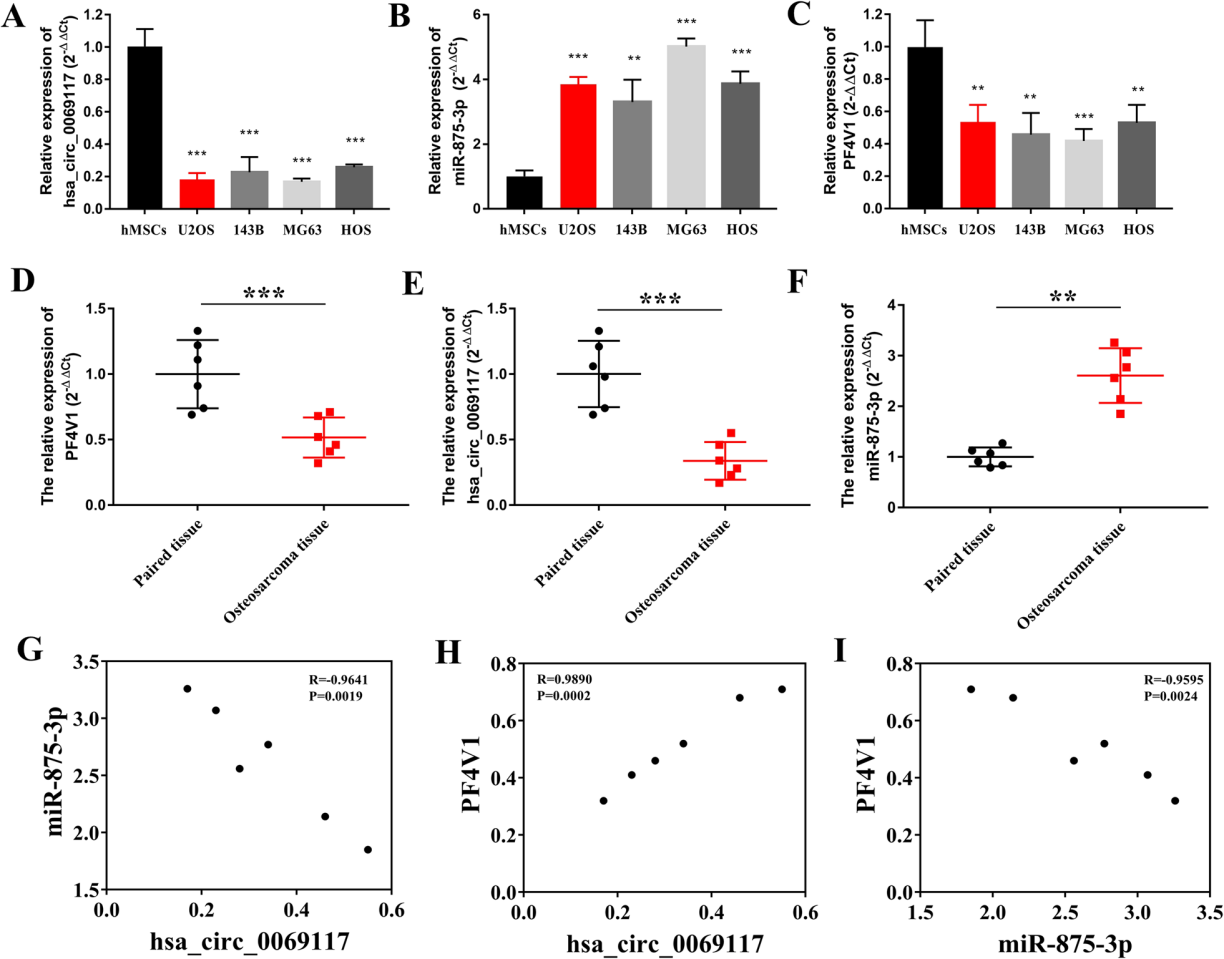
The expression of hsa_circ_0069117 (**A**), miR-875-3p (**B**), and PF4V1 (**C**) in OS clinical samples were validated via qRT-PCR. **D** Negative relationship was observed between hsa_circ_0069117 and miR-875-3p. **E** Negative relationship was observed between miR-875-3p and PF4V1. **F** Positive relationship was observed between hsa_circ_0069117 and PF4V1. The expression of hsa_circ_0069117 (**G**), miR-875-3p (H), and PF4V1 (I) in OS cell lines were validated via qRT-PCR. (n = 3, **p < 0.01; ***p < 0.001)

Next, we detected the expression of hsa_circ_0069117, miR-875-3p, and PF4V1 in four OSCLs. The results indicated that the expression of hsa_circ_0069117 was significantly decreased in OSCLs (Fig. 2A) while the expression of miR-875-3p was significantly increased in OSCLs (Fig. 2B), which was consistent with the results of circRNA (GSE96964) and miRNA (GSE70367) microarrays of OSCLs. The expression of PF4VA was significantly increased in OSCLs (Fig. 2C), which was consistent with the trend of hsa_circ_0069117.

We further validated the expression of hsa_circ_0069117/miR-875-3p/PF4V1 axis in OSTs, which was consistent with that in OSCLs (Fig. 2D–F). Interestingly, the expression of hsa_circ_0069117 was negatively correlated with that of miR-875-3p (Fig. 2G) and positively correlated with that of that of PF4V1 (Fig. 2H). Furthermore, a significant positive relationship was also observed between the expression of miR-875-3p and PF4V1 (Fig. 2I). These results suggest a regulatory relationship between hsa_circ_0069117, miR-875-3p, and PF4V1.

**Fig. 3:**
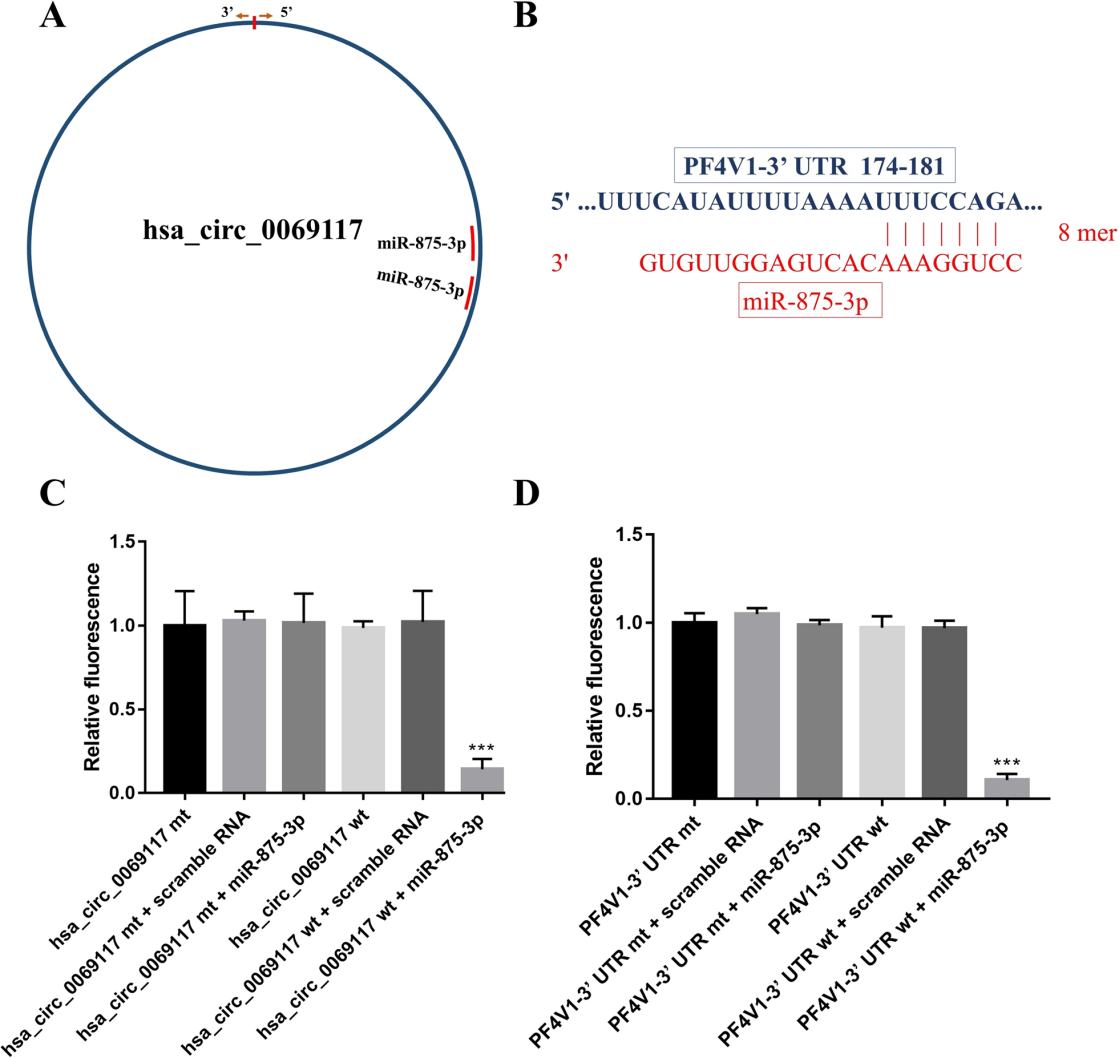
**A** The binding position of miR-875-3p on hsa_circ_0069117. **B** The binding position of miR-875-3p on PF4V1. The luciferase reporter gene assay indicated that miR-875-3p mimics could decrease the fluorescent expression of hsa_circ_0069117-pGL6-miR (**C**) and PF4V1-pGL6-miR (**D**) vectors. (n = 3, ***p < 0.001)

Thereafter, we explored the binding sites of miR-875-3p on hsa_circ_0069117 (Fig. 3A) and PF4V1 (Fig. 3B), which were further validated by luciferase reporter gene assays. The results showed that the luciferase activity of hsa_circ_0069117-pGL6-miR and PF4V1-pGL6-miR vectors was significantly decreased when co-transfected with miR-875-3p mimics (Fig. 3C, D), while the other groups showed no significant changes.

### CircRNA hsa_circ_0069117 could enhance the expression of PF4V1 by sponging miR-875-3p, thereby inhibiting the expression and phosphorylation of ERK1 and AKT

**Fig. 4:**
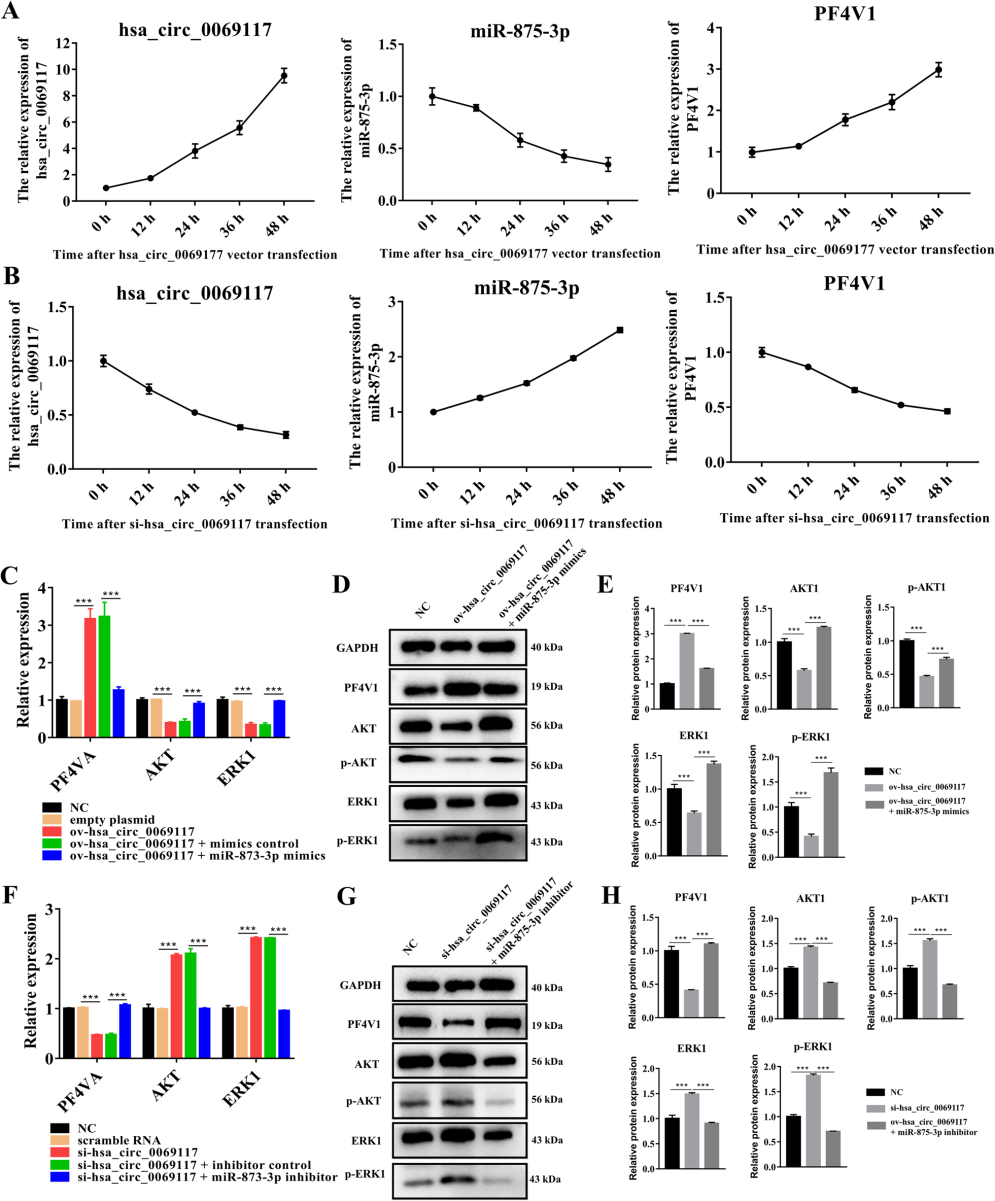
**A** The expression trends of hsa_circ_0069117, miR-875-3p, and PF4V1after hsa_circ_0069117 overexpressing. **B** The expression trends of hsa_circ_0069117, miR-875-3p, and PF4V1 after hsa_circ_0069117 knocking-down. **C** Overexpressing hsa_circ_0069117 could promote the gene expression of PF4V1 but inhibit ERK1 and AKT, which could be reversed by co-transfecting miR-875-3p mimics. **D** and **E** Overexpressing hsa_circ_0069117 could promote the protein expression of PF4V1 but inhibit the protein expression and phosphorylation of ERK1 and AKT, which could also be reversed by co-transfecting miR-875-3p mimics. **F** Knocking-down hsa_circ_0069117 could inhibit the gene expression of PF4V1 but promote ERK1 and AKT, which could be reversed by co-transfecting miR-875-3p inhibitor. **G** and **H** Overexpressing hsa_circ_0069117 could promote the protein expression of PF4V1 but inhibit the protein expression and phosphorylation of ERK1 and AKT, which could also be reversed by co-transfecting miR-875-3p inhibitor. (n = 3, ***p < 0.001)

Gain/loss-of-function assays were performed to verify the effect of hsa_circ_0069117 on miR-875-3p/PF4V1 expression and related pathways. The results showed that overexpressing hsa_circ_0069117 could significantly suppressed the expression of miR-875-3p (Fig. 4A). Inhibition of miR-875-3p by hsa_circ_0069117 promoted the expression of PF4V1 (Fig. 4A). Conversely, suppressing hsa_circ_0069117 significantly upregulated the expression of miR-875-3p thereby inhibiting the expression of PF4V1 (Fig. 4B).

ERK1 and AKT have been reported as downstream targets of PF4V1 [19]. Therefore, we further validated the effect of hsa_circ_0069117/miR-875-3p/PF4V1 on ERK1 and AKT expression. Overexpressing hsa_circ_0069117 significantly promoted the gene and protein expression of PF4V1 but suppressed the gene expression of ERK1 and AKT, while these effects could be reversed by overexpressing miR-875-3p as a rescue method (Fig. 4C). The expression and phosphorylation of ERK1 and AKT protein was consistent with the qRT-PCR results (Fig. 4D, E). Furthermore, knocking down hsa_circ_0069117 significantly inhibited the gene and protein expression of PF4V1 but promoted the gene expression of ERK1 and AKT, while these effects could be reversed by overexpressing miR-875-3p as a rescue method (Fig. 4F). The expression and phosphorylation of ERK1 and AKT protein was consistent with also the qRT-PCR results (Fig. 4G, H). The above results suggested that hsa_circ_0069117 could promote the expression of PF4V1 by sponging miR-875-3p thereby inhibiting the expression and phosphorylation of ERK1 and AKT.

### Overexpressing hsa_circ_0069117 suppresses the proliferation and migration of OS cells

**Fig. 5:**
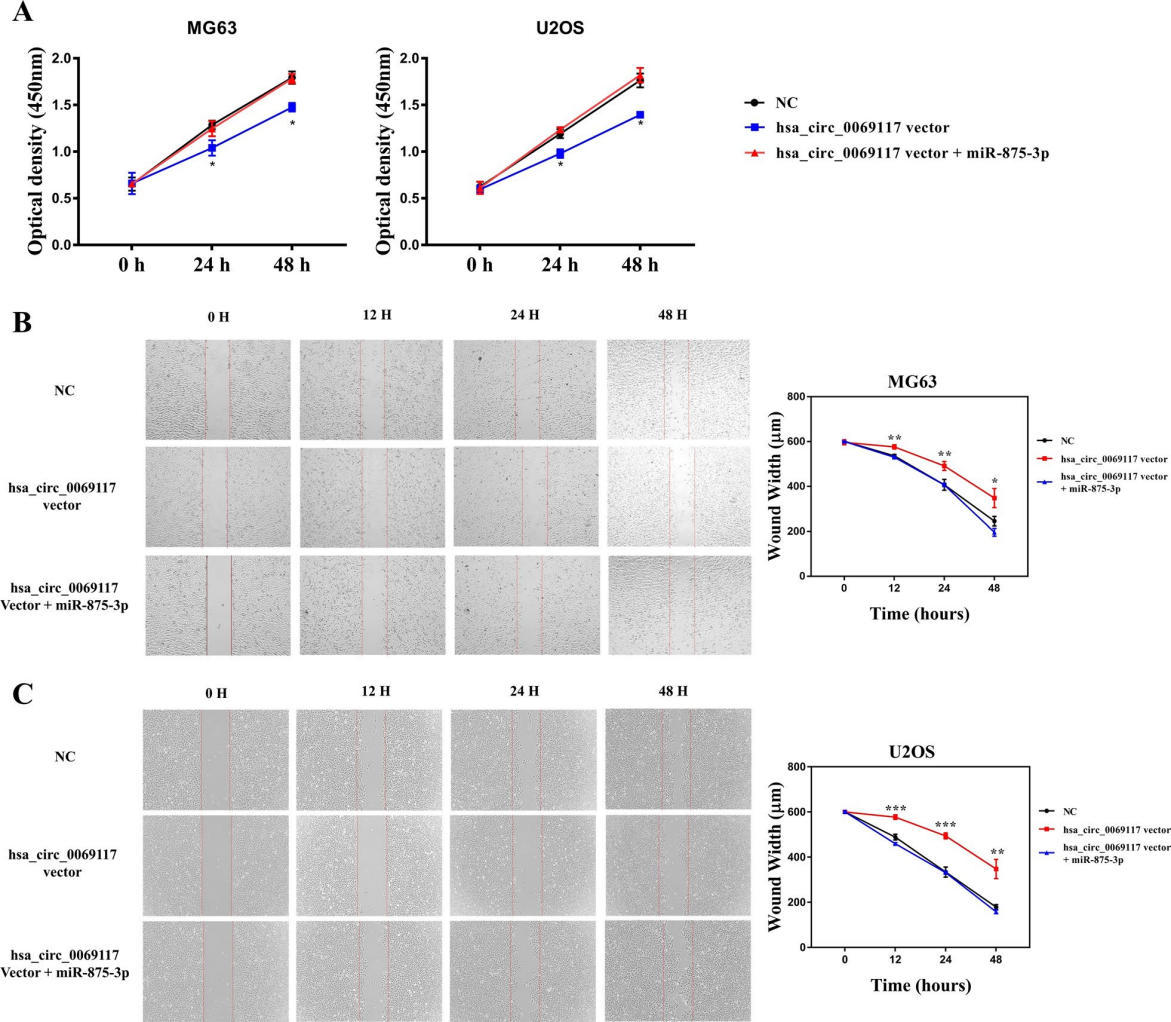
**A** The CCK-8 assay indicated that the proliferation of MG63 and U2OS cell lines was attenuated as hsa_circ_0069117 over-expressed, while it could be reversed by co-transfecting miR-875-3p mimics. **B** The wound healing assay indicated that the migration of MG63 and U2OS cell lines was attenuated as hsa_circ_0069117 over-expressed, while it could also be reversed by co-transfecting miR-875-3p mimics. (n = 3, *p < 0.05; **p < 0.01; ***p < 0.001)

Subsequently, we validated the effect of hsa_circ_0069117 on the proliferation and migration of OS cells. As hsa_circ_0069117 was most remarkably decreased in MG63 and U2OS cell lines, we performed CCK-8 and wound healing assays on MG63 and U2OS cell lines after hsa_circ_0069117 overexpression. The results indicated that the proliferation (Fig. 5A) and migration (Fig. 5B, C) of MG63 and U2OS cell lines were attenuated as hsa_circ_0069117 over-expressed. However, the attenuated effect of hsa_circ_0069117 on OS cell proliferation and migration could also be reversed by co-transfection with miR-875-3p mimics (Fig. 5B, C).

## Discussion

Recent years have witnessed the great progression of circRNA research in functioning as a gene expression regulator in various diseases [20–22]. Several studies have revealed the dysregulation of circRNAs in OS, which affects many critical processes of OS, including proliferation, invasion, and metastasis [23–25]. Zhang et al. found that circ-0002052 could enhance the activation of Wnt/β-catenin and then stimulate the progression of OS via sponging miR-382 [23]. Yan et al. have reported that CircRNA PVT1 could promote metastasis via circPVT1/miR-526b/FOXC2 axis [26]. Yang et al. found circ_0001105 was expressed at low levels in OS cells, and their further study indicated it could suppress OS progression by regulating the miR-766/YTHDF2 axis [27]. Such preliminary studies indicated that different circRNAs could function as enhancers or suppressors of OS.

**Fig. 6:**
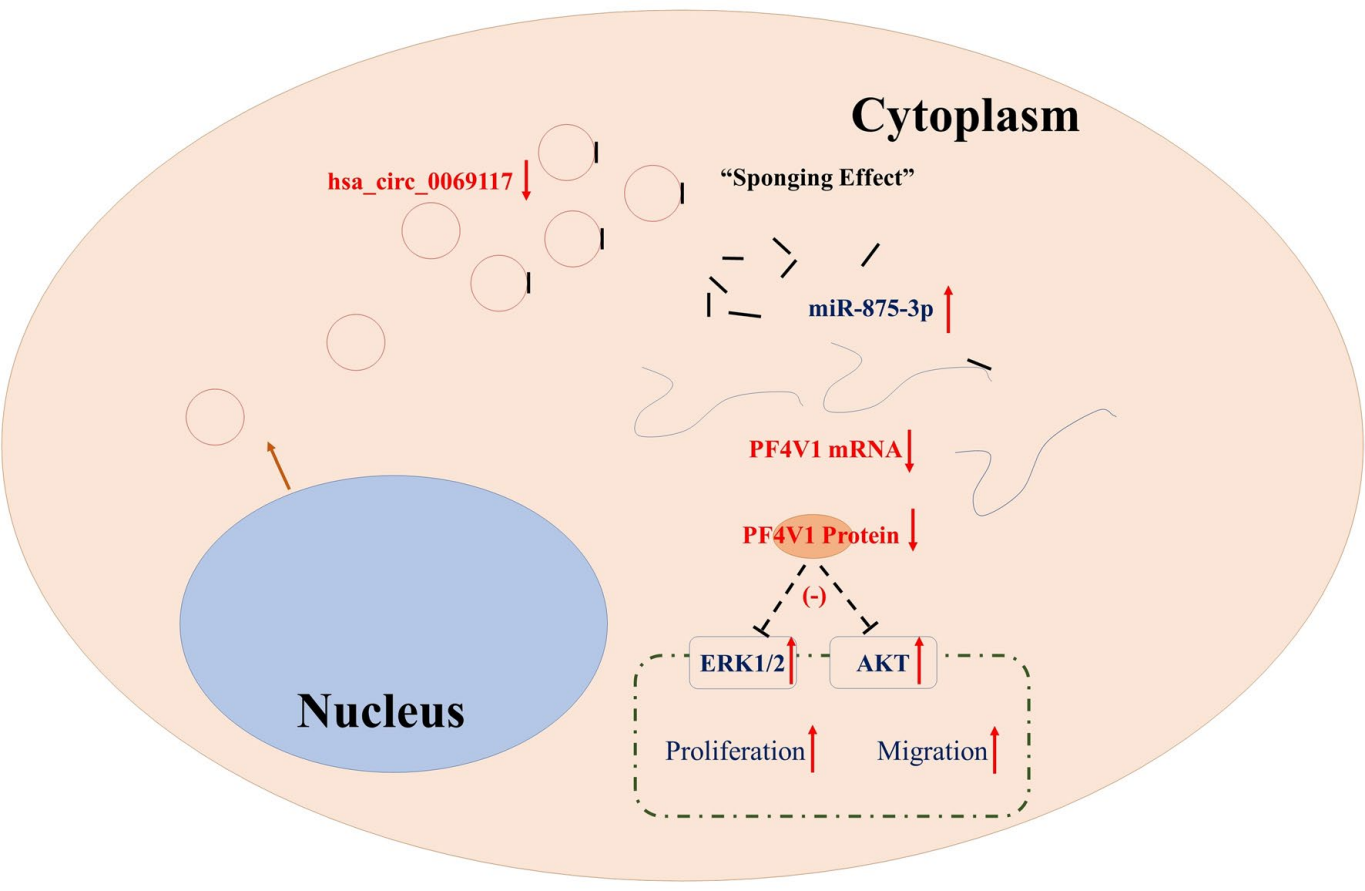
The regulatory effect of hsa_circ_0069117 on OS cell proliferation and migration

In present study, hsa_circ_0069117 was found to be the most markedly dysregulated in OS cells. However, the regulatory effect of hsa_circ_0069117 in malignant diseases, including OS, remains unclear. To further investigate the potential regulatory roles of hsa_circ_0069117 in OS, we predicted its potential target miRNAs. Sixty miRNAs were predicted to be targets of hsa_circ_0069117. By co-analyzing with the miRNA expression profile in OS, we found that only miR-875-3p was differentially expressed. MiRNA-875-3p has already been reported as an oncogene regulator by targeting PF4V1 [19]. PF4V1, also known as CXCL4L1, suppresses cell proliferation, migration, and invasion in various cancer by targeting ERK1 and AKT [28–30]. AKT/ERK pathway is critical intracellular signal transduction cascades, regulating cell proliferation and migration [31]. Previous studies indicated that AKT/ERK pathway could enhance the activity of osteosarcoma cell proliferation and migration and promoted osteosarcoma progression [32]. Based on these findings, we hypothesized that hsa_circ_0069117 could regulate the progress of OS via the miR-875-3p/PF4V1 axis (Fig. 6).

We constructed several lines to confirm our hypotheses [13]. First, we validated the expression of hsa_circ_0069117, miR-875-3p, and PF4V1 in four OSCLs and OSTs. The results showed that the expression of hsa_circ_0069117 and PF4V1 was decreased while the expression of miR-875-3p was increased, which was in line with our prediction. Second, we confirmed the binding sites of miR-875-3p on hsa_circ_0069117 and PF4V1 using luciferase reporter gene assay. Third, an in vitro functional verification assay of hsa_circ_0069117 was performed and the results showed that hsa_circ_0069117 could significantly suppress the expression of miR-875-3p and promote the expression of PF4V1. High level of PF4V1 could suppress the expression and phosphorylation of ERK1 and AKT [33, 34]. Fourth, the proliferation and migration of OSCLs could be attenuated by overexpressing hsa_circ_0069117 and reversed by co-transfection with miR-875-3p mimics.

In summary, we conducted this study to elucidate the regulatory effect of hsa_circ_0069117 on OS progression. We identified and further confirmed that miR-875-3p/PF4V1 axis was the target of hsa_circ_0069117. Over-expression of hsa_circ_0069117 substantially attenuated the proliferation and migration of OS cells. Our study provides novel and reliable molecular target for the diagnosis and therapy of patients with OS.

## Data Availability

All data generated during this study are available from the corresponding author.

## Abbreviations

AKT1: Protein kinase B
ATCC: American type culture collection
CCK8: Cell counting kit-8
circRNA: Circular RNA
ERK1: Extracellular signal-regulated kinase 1
FUNRICH: Functional enrichment analysis tool
GEO: Gene expression omnibus
ncRNA: Non-coding RNA
OS: Osteosarcoma
OSCL: Human osteosarcoma cell lines
OST: Osteosarcoma tissues
PF4V1: Platelet factor 4 variant 1
qRT-PCR: Quantitative real-time PCR
TBC1D14: TBC1 domain family member 14
